# Impact of RAASIs on Potassium and Mortality in a Large Cohort of Hemodialysis Patients: Practical Excursus and Comparison Between Traditional Statistics and Machine Learning

**DOI:** 10.3390/jcm15134928

**Published:** 2026-06-25

**Authors:** Vincenzo Calabrese, Maria Rita Stancanelli, Maria Eva Sberna, Giovanni Taverna, Giulio Geraci, Valeria Cernaro, Domenico Santoro

**Affiliations:** 1 Department of Medicine and Surgery, University of Enna “Kore”, 94100 Enna, Italy; giulio.geraci@unikore.it; 2 Unit of Nephrology and Dialysis, Department of Medicine and Surgery, Hospital of Enna “Umberto I”, 94100 Enna, Italy; mariarita.stancanelli@gmail.com (M.R.S.); mariaevasberna@gmail.com (M.E.S.); 3 PhD Program in Translation Specialistic Medicine “G.B. Morgagni”, Curriculum “Cardiovascular Sciences”, Department of Cardiac, Thoracic, and Vascular Sciences and Public Health, University of Padua, 35128 Padua, Italy; giovanni.taverna@studenti.unipd.it; 4 Unit of Nephrology and Dialysis, Department of Clinical and Experimental Medicine, A.O.U. “G. Martino”, University of Messina, 98122 Messina, Italy; vcernaro@unime.it (V.C.); dsantoro@unime.it (D.S.)

**Keywords:** dialysis, longitudinal analysis, machine learning, mortality, RAASIs, Random Forest, serum potassium

## Abstract

**Background:** The 2022 Kidney Disease: Improving Global Outcomes (KDIGO) guidelines suggest the use of Renin–angiotensin–aldosterone system inhibitors (RAASIs) in chronic Kidney Disease (CKD) stages IV–V, in contrast to the 2012 KDIGO guidelines, which discouraged it. This study aims to assess the impact of RAASIs on kalemia and mortality in a large sample of dialysis patients, where longitudinal data remain scarce, comparing traditional statistical methods with machine learning (ML) algorithms. **Methods:** This observational longitudinal analysis included 4764 hemodialysis (HD) patients from the Sicilian Registry of Nephrology, Dialysis and Transplantation, with a total of 56,964 longitudinal measurements. We evaluated the impact of RAASIs on serum potassium levels and all-cause mortality in the dialysis setting, comparing traditional statistics and ML. Linear Mixed Models (LMM) and Cox models with mixed effects were used for longitudinal and survival analyses. These were compared with ML approaches, including Random Forest (RF) for potassium variability and Lasso-regularized models for mortality, using four-fold cross-validation. **Results:** The study included 4764 patients, of whom 1207 (25%) were treated with RAASis. The mean age was 66 ± 15 years, 62% were male, 33% were diabetic, and a history of arterial hypertension was reported in 74% of patients. Hyperkalaemia at baseline was present in 1848 patients. The longitudinal model showed a statistically significant increase in kalemia [adjβ = 0.10 mmol/L, 95%CI 0.05/0.15, *p* < 0.001], but it was clinically negligible. Indeed, RF did not detect RAASIS as a relevant variable. Association between RAASIs and mortality was not detected either with Cox or ML models. Furthermore, the RF model outperformed traditional LMMs in explaining total potassium variability (56% vs. 43%). **Conclusions:** RAASI therapy in HD patients is associated with a minimal, non-clinically significant increase in serum potassium and does not impact all-cause mortality. The integration of ML reinforces the robustness of these findings, supporting the safety of RAASIs in the dialysis setting.

## 1. Introduction

Renin–angiotensin–aldosterone-system inhibitors (RAASIs) are widely used anti-hypertensive drugs, but they are often withdrawn when eGFR is below 30 mL/min/1.73 mq. This happens because aldosterone is responsible for sodium reabsorption in the distal tubule and collecting duct, through the excretion of hydrogen and potassium ions, and leads to an increase in potassium levels [1]. For this reason, considering the high risk of hyperkalemia in patients with advanced chronic kidney diseases (CKD), the 2012 KDIGO Guidelines advised not to prescribe RAASIs in patients with an eGFR less than 30 mL/min/m2. Despite these recommendations, recent studies suggested a potential cardioprotective and nephroprotective action of RAASIs and a reduction in comorbidity related to RAASI use, also in patients with severe CKD [2], resulting in a substantial change in the new KDIGO guidelines, which allow their use in CKD patients, regardless of the severity [3].

A recent large longitudinal study evaluated the impact of RAASIs on serum potassium and on all-cause mortality in dialysis patients [4], which did not show a significant impact of RAASIs on serum potassium or mortality. Furthermore, this study details no difference in hyperkalemia events in the RAASIS group compared to patients who did not take RAASIs, and it specifies the percentage of treatment changes. It used advanced statistical analysis, but we aim to compare these results with machine learning approaches to improve the strength and internal validity of these results.

Machine learning (ML) encompasses a diverse set of algorithms that enable computers to learn from data without explicit programming. ML methods, such as CART and Random Forest, are used in different real-world examples, from medicine to finance or marketing [5].

These methods can be categorized into two major groups: supervised learning and unsupervised learning [6]. Supervised learning involves learning from labelled data, where the algorithm is provided with both input features and the corresponding output. Among these, we retrieved Classification and Regression Trees (CART), Random Forest (RF) and Neural Network (NN).

CART is a versatile technique that partitions the predictor space into smaller, more homogenous regions [7], maximizing the purity of the resulting nodes. This approach allows CART to effectively capture non-linear relationships between predictors and the outcome and can be used to both categorical and continuous variables, enabling both classification and regression tasks [7].

The output of CART is a decision tree—a visual representation of the decision-making process—providing a clear and concise visual representation of the model and facilitating easy interpretation and communication of results.

Random Forest is an ensemble learning method that is derived from decision trees. It constructs multiple decision trees during the training process, each trained on a different bootstrap sample of the data and using a random subset of features for each split [8,9].

By aggregating the predictions from multiple trees, Random Forest effectively reduces overfitting more than single-decision trees. Indeed, similar to the CART algorithm, it partitions samples into more homogeneous subsamples but uses a random subset of features for each split [10]. This ensemble approach also improves the model’s generalization ability and enhances its robustness to noise in the data [11].

Random Forest provides a measure of feature importance, indicating the relative contribution of each predictor to the model’s predictive accuracy [12]. This information can be valuable for identifying the most influential factors and gaining deeper insights into the underlying data.

In a previous analysis, we evaluated the impact of RAASIs on serum potassium and mortality in a large sample of dialysis patients retrieved by the Sicilian Registry of Nephrology, Dialysis, and Transplantation [4]. In the present study, we will use those results as examples to compare the traditional statistical analysis with the machine learning approaches.

## 2. Materials and Methods

The study conforms with the Italian Data Protection Authority guidelines and is in agreement with the Helsinki Declaration. We analyzed data from the Sicilian Registry of Nephrology, Dialysis and Transplantation (SRNDT), a collection of regional data, instituted in 2008 by regional laws. The Sicilian registry was established with the aim of collecting data on CKD patients for scientific purposes (decree 03423/08). Informed consent is requested from all patients whose data are entered into the registry. However, as specified in Art. 1, no formal approval from ethical committees is needed to analyze data, as they are available only anonymously.

Demographic and clinical characteristics, dialysis details, comorbidities, and mortality of patients undergoing chronic renal replacement therapy, as well as information regarding renal transplantation in Sicily, were collected on the REGDIAL web platform (Cooperativa EDP La Traccia, Matera, Italy). Data were extracted from the SRNDT in accordance with ethical standards and respect for privacy.

### 2.1. Study Population and Laboratory Data

In the previously published analysis [4], we included hemodialysis patients entered into the Sicilian Registry of Nephrology, Dialysis, and Transplantation from 1 January 2018 to 31 December 2020. As reported in the published cohort analysis, 6451 patients were retrieved [13]. Among them, 1687 patients were excluded (1220 for lacking the potassium measurement and 467 for missing RAASIs information), for a total of 4764 included patients (for a total of 56,964 measures) (Supplementary Figure S1).

RAASI assumptions and serum potassium measurements were collected for each patient since the start of the dialysis treatment, as well as the main demographic, somatometric, clinical, and biochemical characteristics of the study population. Only patients with no missing values for these variables were included, with the median RAASI and serum potassium measurements of 10 [4–28] visits.

Median follow-up was 38 [13–83] months, for a total of 10 [4–28] visits. Details regarding drug history and dialysis treatment were reported in the previous article.

### 2.2. Data Collection

Laboratory and clinical data were collected locally from the Register referents and then entered into the platform. Laboratory data included serum phosphate, haemoglobin, C-reactive protein, iron, transferrin, ferritin, potassium, calcium, intact PTH, albumin, glucose, triglycerides, cholesterol, bicarbonate, alkaline phosphatase, fractional urea clearance (Kt/V), and β2-microglobulin. Clinical data included blood pressure levels, residual diuresis, previous comorbidities (dementia, hemiplegia, liver disease, history of arterial hypertension, vascular disease, chronic obstructive pulmonary disease (COPD) malignancy with/without metastasis, heart failure, psychiatric disease, dyslipidemia, prostatic hypertrophy), as well as pharmacological treatment such as anti-hypertensives, folic acid, calcium carbonate, cholecalciferol, insulin, aspirin, allopurinol, phosphorous binder, calcium-mimetics, cortisone, erythropoiesis-stimulating factors (ESA), iron supplementation, immunosuppressive treatment, proton pump inhibitors, paricalcitol, and vitamin B12. Details of this Registry are described elsewhere [14].

### 2.3. Statistical Analysis

#### 2.3.1. Traditional Statistics

The distribution of variables was investigated using the Kolmogorov–Smirnov test followed by graphic evaluation. Baseline data were described as mean ± standard deviation, median and interquartile range, or proportion, as appropriate. Serum potassium, as well as the quantitative confounders, were included in the analysis as continuous variables (Table 1).

The proportion of missing data differed for each variable. Specifically, KT/V was not available in 22% of measurements, BMI in 8%, albumin in 29%, PTH in 41%, haemoglobin in7%, serum calcium in 4%, serum phosphate in 4%, ferritin in 42%, HCO3 in 54%, the systolic pressure in 11%, CRP in 59% of measures, respectively. Other variables included in the multivariate models had less than 0.01% missing data.

Missing values were related neither to the center that provided the data nor to specific characteristics of patients, so were considered at random. As mixed-effects models are well equipped to handle missing (at random) response data if estimated using likelihood methods, we did not impute or recover them in the previous study.

In the traditional statistical analysis, the longitudinal association between RAASIs intake and serum potassium was analyzed using univariate and multivariate Linear Mixed Models (LMM). In this analysis, all variables related to RAASI intake and serum potassium with a *p*-value < 0.2 were selected as potential confounders (age, sex, albumin, hypertension, calcium receptor blocker, COPD, CRP, diabetes, diuresis, serum iron, heart failure, serum phosphate, Tsat, visit and KT/V).

We tested various univariate and multivariate models (one univariate random intercept LMM model, two univariate LMM models performing both random slope and random intercept, one multivariate random intercept model using ID as a random intercept variable, two multivariate LMM models with random slope variables, and three multivariate LMM models with both random slope and random intercept). The details can be retrieved from the previous article.

Similarly, the survival models were computed by testing various models. Specifically, we computed one univariate Cox model without a random effect, one univariate Cox model with mixed effects, and two multivariate Cox models with mixed effects. Both for the LMM models and for the Cox models, the best-fitted models were chosen according to the Akaike criteria.

#### 2.3.2. Machine Learning Approach

In the machine learning approach, confounders were chosen using a Random Forest approach. Due to the Random Forest approach requiring no missing data, an RF imputation was applied to our database for the Machine learning approach. The whole dataset was split into a training set and a test set, with a proportion of 75% and 25%, respectively.

**Figure 1:**
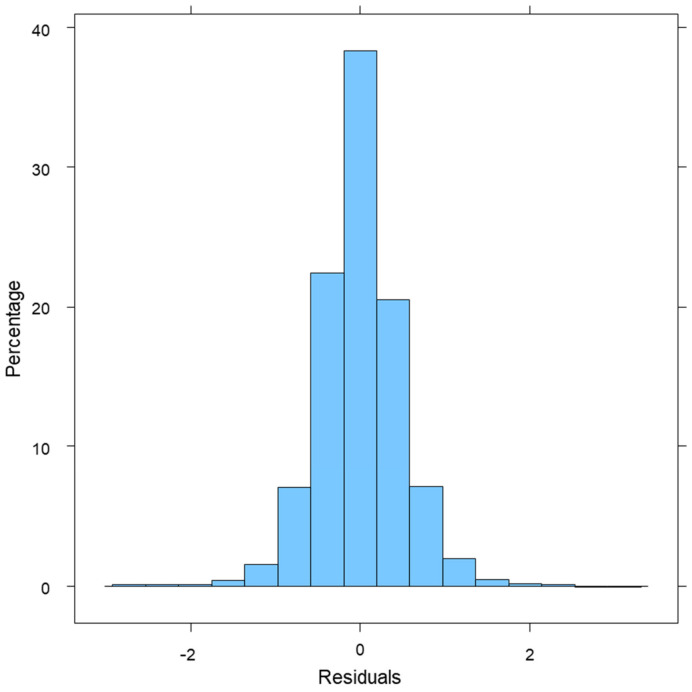
Residual distribution of the linear mixed-model analysis.

In keeping with the longitudinal structure of our data, we started our Random Forest approach by utilizing an LMM model using serum potassium as the dependent variable and all the variables included in the dataset as independent variables. The residuals were saved and used as the dependent variable of our Random Forest approaches [15,16]. The mean of the residuals was closest to zero (−1.92 × 10^−14^), with a Gaussian distribution [17] (Figure 1).

To optimize the Random Forest (RF) model and prevent overfitting, the number of trees in the forest was set to 500. The cross-validated Random Forest approach has been trained with the R-studio function “caret::train”, splitting the database into four folds to optimize hyperparameters and to ensure robust performance evaluation. The dataset was randomly divided into four folds of equal size. The number of variables randomly sampled as candidates at each split was identified as 20, after considering all the available options from 2 to (number of variables −1) through the “Tunegrid” function. The final model’s stability was assessed by evaluating the minimal depth of the trees and ensuring that the forest’s complexity was balanced against its predictive power. Stratified sampling ensured that the distribution of the target variable was maintained across all folds. The model was trained on three folds and tested on the remaining fold, repeating this procedure four times—one for each fold for the test. The performance metrics Root Mean Squared Error (RMSE) and Mean Absolute Error (MAE) were calculated for each fold and subsequently averaged to obtain an overall estimate of the model’s performance.

Thus, the final Random Forest approach was trained with an R-studio function “randomForest” with a number of trees of 500, and a number of variables taken for the analysis for each split of 20. For each variable, the percentage MSE increase was computed, and the variables with an %IncMSE upper than the mean were included in a multivariate LMM that used serum potassium as the dependent variable, to compare this model to the traditional model using the identical dependent variable and identical dataset.

The survival model was developed as a cross-validated survival analysis using the R-studio function “cv.glmnet”. Due to the high number of variables, we applied lasso regularization and chose the lambda value that makes the model as explainable as possible (a minor number of covariates included) but with a standard error of less than 1 standard deviation from the best model. In the lasso regularization of the survival model, all the variables with a coefficient close to 0 were deleted. Due to this model not considering mixed effects, the variables with a valid coefficient (31 variables) were included in the Cox analysis with mixed effects.

## 3. Results

### 3.1. Impact of RAASIs on Serum Potassium

Traditional statistical analysis retrieved 15 covariates to include in the multivariate model (RAASIs, age, sex, albumin, arterial hypertension, use of calcium antagonist, COPD, CRP, diabetes, diuresis, serum iron, heart failure, serum phosphate, Tsat, KT/V). This model showed a slightly significant impact of RAASIs on kalemia, of about 0.1 mmol/L [adjβ = 0.10, 95%CI 0.05/0.15, *p* < 0.001]. The fixed effects of this model explain 43% of the variability. This means that 43% of the potassium values were explained by the variable included in this multivariate model (Table 2).

Severe hyperkalemia higher than 6.5 mmol/L was manifested at least once in 872 patients (15.74%), without significant differences between the RAASIS group and no-RAASIS group (15.8% vs. 15.7%, *p* = 0.965). The multivariate model showed significant associations between hyperkalemia and albumin, diabetes, phosphate, Tsat, COPD, dialytic age, ASA, and diuresis, but not with RAASI use.

**Figure 2:**
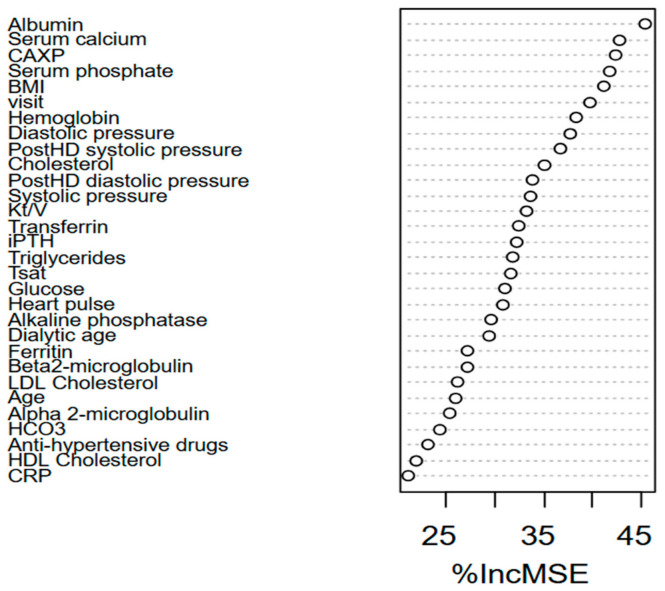
Importance of impact on serum potassium detected by the Random Forest model.

The basic Random Forest model gave us a panel of % of MSE increases for each variable. The variables with a %IncMSE higher than the mean were sex, KTV, BMI, iPTH, albumin, HDL cholesterol, triglycerides, hemoglobin, beta 2 microglobulin, serum calcium, ferritin, total cholesterol, CRP, serum glucose, serum phosphate, LDL cholesterol, serum bicarbonate, calcium-phosphate product, pre-HD systolic pressure, pre-HD diastolic pressure, alkaline phosphatase, transferrin saturation, post-HD systolic pressure, post-HD diastolic pressure, heart pulse, diuresis, age, dialytic age, antiaggregant use, insulin treatment, erythropoiesis-stimulating agents, phosphate binders, serum iron, diabetes, COPD, and calcitriol (Figure 2).

We should highlight that all the variables related to kalemia with a *p*-value less than 0.2, which were thus included in the traditional multivariate LMM model, have been retrieved in the Random Forest approach. Among the variables included in the multivariate model but with no significant association in multivariate analysis, only the KTV was retrieved by the Random Forest approach because it had an impact on kalemia. Indeed, KTV had the coefficient nearest to 0 (beta: −0.083, 95%CI −0.25/0.08, *p* = 0.329) in the multivariate LMM, but RF detected a high impact of KT/V on serum potassium. This can be clinically explained by a nonlinear association between these two variables, which does not represent a limit in the RF approach.

In our model, the %IncMSE of RAASIs was 13.22, with a %IncMSE mean of 14.38. Thus, RAASIs seem not to have an impact on kalemia. Indeed, traditional statistics showed a significant association, but with a coefficient of 0.1 mmol/L, which was not clinically significant.

Furthermore, the RF model explains 53% of the variability.

The cross-validated Random Forest approach detected 20 as the best number of variables chosen for each split. This model is comparable with the basic Random Forest approach, with irrelevant differences in the %IncMSE.

Specifically, the selected variables were sex, KTV, BMI, iPTH, albumin, HDL cholesterol, triglycerides, hemoglobin, beta 2 microglobulin, serum calcium, ferritin, total cholesterol, CRP, serum glucose, serum phosphate, LDL cholesterol, serum bicarbonate, calcium-phosphate product, pre-HD systolic pressure, pre-HD diastolic pressure, alkaline phosphatase, transferrin saturation, post-HD systolic pressure, post-HD diastolic pressure, heart pulse, age, dialytic age, antiaggregant use, insulin treatment, alloputrinol, PPI, erythropoiesis-stimulating agents, phosphate binders, serum iron, diabetes, and calcitriol.

The only differences were retrieved in diuresis and COPD, which were not detected in the CV model, and allopurinol and PPI were detected in the CV model. The CV RF model explains 56% of the variability.

### 3.2. Impact of RAASIs on Mortality

In the traditional analysis, neither the univariate Cox model with mixed effects nor the multivariate Cox model with mixed effects showed the significant impact of RAASI intake on mortality (HR = 0.82, 95%CI 0.66/1.03, *p* = 0.09 and HR = 0.76, 95%CI 0.43/1.30, *p* = 0.31, respectively). The variables included in the multivariate analysis are reported in Table 3.

As specified in the methods section, the machine learning approach selected variables that had a relevant impact on mortality. Specifically, our model selected arterial hypertension, visit, age, diuresis, serum potassium, diabetes, CRP, hemoglobin, arrhythmia, alkaline phosphatase, phosphate binders, KTV, heart failure, total cholesterol, metastasis, diabetes, vascular disease, transferrin saturation, albumin, insulin treatment, serum glucose, Paracalcitolo, B12 Vitamin treatment, liver disease, cinacalcet treatment, anemia, COPD, BMI, HDL cholesterol. RAASI use is not selected by our cross-validated model, according to traditional statistical analysis, which did not retrieve the statistical impact of RAASIs use on mortality.

The machine learning approach retrieved all the variables related to mortality with a *p*-value less than 0.2 using traditional statistics, excluding serum phosphate and antiaggregant. Although this seems to disagree with the traditional Cox model, we should highlight that antiaggregant use had the HR closest to 1 (HR: 0.975, 95% CI 0.6/1.6) in the multivariate model, which represents no significant impact when the model includes all related variables. Furthermore, although serum phosphate was not retrieved, phosphate binders were, and these drugs are clinically related to serum phosphate.

## 4. Discussion

Our analysis compared traditional statistics to machine learning approaches to evaluating the impact of RAASIs on serum potassium and mortality in a cohort of dialysis patients.

In traditional analysis, the allocation was represented by the use of RAASIs. Consequently, the confounding search is derived from this variable. RAASIs seemed to be slightly related to serum potassium concentration in our sample. Although this relationship was statistically significant, its coefficient was not clinically relevant.

The RF approach did not reveal a significant association between RAASI and kalemia, but its impact was just below the mean. Despite this apparent disagreement, these two results are concordant. Indeed, the LMM model showed a very light coefficient with a potassium increase of 0.1 mmol/L in patients who used RAASIs, and this has no clinical relevance. Thus, while the traditional LMM indicated a statistically significant association between RAASIs and potassium, its exclusion from the most relevant features in the Random Forest model confirms that this statistical significance does not translate into clinical relevance, as other physiological and dialytic factors play a far more predominant role in potassium homeostasis. Furthermore, the fact that Random Forest achieved a significantly higher R2 (0.56) compared to the Linear Mixed Model (0.43) suggests that the relationship between clinical variables and serum potassium in hemodialysis is characterized by complex, non-linear interactions that traditional linear statistics fail to fully capture, and forces the hypothesis that RAASIs use is not strongly implied in the genesis of hyperkalemia in this setting as well as dismitting them.

Furthermore, many other variables have been identified as related to serum potassium, such as insulin or erythropoiesis-stimulating agents use, aspirin use, or serum bicarbonate values. All of them have clinical associations with serum potassium, and this allows us to consider these results plausible. Notably, they were detected by the RF approach in a model with a higher explained portion of variability than traditional analysis.

Although allopurinol and PPI disagreed between RF and CV RF, it can be easily explained. In the CV RF approach, the mean value of %IncMSE was 14.5. Allopurinol and PPI had a %IncMSE of 14.7 and 14.9, respectively. These values are closest to the minimum selected value when the range of selected variables had a value from 14.5 to 50.09. It reflects a slight impact of these two variables, which cannot be detected with the little differences between the two methods. Indeed, in the RF approach, their %IncMSE was close to the minimum selected value (13.7 and 14.1, respectively).

However, it is important to note that our result differs from that of Movilli et al., who reported in 112 anuric hemodialysis patients,19% of patients developing severe hyperkalemia requiring RAASIs discontinuation [18]. This discrepancy can be explained by different population characteristics. Indeed, in our study, 33–48% of patients maintained residual diuresis (Table 1), whereas Movilli studied exclusively anuric patients. Residual diuresis is a critical protective factor against hyperkalemia, as even small urine volumes contribute significantly to potassium excretion. This suggests that RAASIs may be particularly safe in dialysis patients with residual diuresis, while requiring closer monitoring in anuric patients [19].

The practical implications of our findings align with recent KDIGO 2024 guidelines, which recommend continuing RAASIs even with eGFR < 15 mL/min/1.73 m^2^, reserving dose reduction or discontinuation only as a last resort in case of uncontrolled hyperkalemia despite medical treatment. Indeed, the strategies to mitigate hyperkalemia without discontinuing RAASIs include the optimisation of dialysate potassium concentration, the nutritional counselling to avoid potassium-containing salt substitutes, the correction of metabolic acidosis with bicarbonate, the use of diuretics in patients with residual diuresis, and the use of the potassium binders, which have demonstrated efficacy in maintaining normokalemia while allowing RAASIs continuation for up to 12 months.

The machine learning survival model showed no impact of RAASIs on mortality, according to the traditional Cox model. However, machine learning survival models have been able to detect more variables related to mortality than traditional methods, which are not detected by these latter. In keeping with the previous Random Forest analysis on serum potassium, all of them have an important clinical association with mortality (e.g., BMI, dyslipidemia, anaemia, residual diuresis).

In addition to comparing the two methods, these analyses improve the strength and the internal validity of the previous hypothesis of safety in the use of RAASIs in dialysis patients. Indeed, although in the traditional statistical analysis, we performed many models yet to improve internal validity, all with similar results, the strength of the analysis has been improved due to RF confirming the absence of clinical impact of RAASIs on potassium and on mortality.

Our findings provide a strong suggestion that the routine discontinuation or avoidance of RAASIs in hemodialysis patients, driven by the fear of hyperkalemia, is not clinically justified. Given the negligible impact on potassium levels and the safety on mortality, clinicians should avoid discontinuing RAASIs in dialysis patients, which would reduce the potential cardiovascular benefits of them.

Although the U-shaped relation between serum potassium and mortality is known, the interest in the impact of potassium variability is still increasing. Indeed, whereas the serum potassium seems not to be significantly impacted by the RAASIs use according to our analysis, the potassium variability during the dialysis treatment confirms its worsening role on the dialysis survival [20,21].

This aligns with recent evidence in AKI and severe CKD [22,23], which demonstrates that discontinuing these agents after an acute decline in kidney function does not lower the risk of all-cause mortality and progression to end-stage kidney disease.

As specified in the previous article [4], our study included only patients from one Italian region with a high prevalence of Caucasians, and this impacts its generalizability.

### 4.1. The Usefulness of Machine Learning

Random forest gives us a list of important variables to the model and helps identify potential overfitting. However, interpreting the importance of variables in complex RF models with numerous trees and multiple validation steps can be challenging [24,25]. The consistency of results across both the traditional Cox proportional hazards model and the Lasso-regularised ML model, both failing to show an association between RAASIs and mortality, provides a high level of confidence in the safety profile of these drugs in a dialysis population.

Furthermore, the selected variables from the Random Forest approach were validated in a new Linear Mixed Model using serum potassium as the outcome variable. This validation model achieved an R^2^ of 56%, compared to 43% in the traditional confounder-based model. This two-stage approach (RF for variable selection, LMM for validation) combines the advantages of machine learning in capturing non-linear relationships with the interpretability of linear models, while avoiding the circularity of using residuals as final outcomes.

The superior performance of Random Forest (R^2^ 0.56 vs. 0.43) likely stems from its ability to capture non-linear interactions between variables that traditional models overlook. For instance, the impact of RAASIs might be conditional on the other factors visible in Figure 2. By considering these multi-dimensional interactions, the ML algorithm filtered the RAASI effect as not clinically relevant, whereas the LMM captured its statistically significant effect, even though the clinical impact was not relevant.

Furthermore, the Lasso regularization approach of the survival model effectively addressed potential multicollinearity among clinical predictors, and it isolated the most parsimonious set of mortality risk factors, among which RAASI treatment did not emerge as a significant feature. This increases the robustness and interpretability of our study.

### 4.2. Limitations of Machine Learning

The important limitations of machine learning methods can be summed up as follows: interpretability, data requirements, and overfitting. Indeed, it is not easy to interpret the specific mechanisms used in the machine learning approach to compute these relationships, and machine learning algorithms often require large datasets to achieve optimal performance [26].

Furthermore, the risk of overfitting remains, despite techniques such as cross-validation, especially with complex models and limited data, and they require the correct training of the model and an accurate test analysis, which needs to be well interpreted [27].

Ultimately, in some cases, the decision-making process within complex machine learning models can be difficult to fully understand, limiting interpretability. This remains true even though it happens more in neural networks or deep learning, which include the black box in the process, than in Random Forest models [28].

This study provides valuable insights into the comparative strengths of traditional statistical methods and machine learning approaches in the analysis of health data. Indeed, it is crucial to investigate the optimal integration of these approaches in the data analysis to enhance the understanding of disease physiopathology and ameliorate patient management.

## 5. Conclusions

In conclusion, in our analysis, RAASI, with a %IncMSE of 13.22, appears to have a very low impact on kalemia, which has no clinical significance. This agrees with the very low coefficient, although statistically significant, of the LME model including confounders as covariates. Furthermore, the model including covariates selected by RF has an explained variance of 53% (higher than the 43% of the model selected with the confounder search).

Similarly, the analysis generated by including variables selected with CV.glmnet provides us with a model with significantly lower variance than the traditional analysis. Despite the very low quality of the data due to the non-homogeneous recording, ML allows us to detect better models, even though they are not easy to understand. These differences between ML and traditional statistics exist because ML is capable of detecting even non-linear dependencies between variables, which can be hidden from traditional statistics.

According to both models, RAASIs have no impact on mortality, which is why the suspension of RAASIs in dialysis patients deserves further specific evaluation through RCTs. Furthermore, the diffusion and the knowledge of machine learning should be improved among clinicians to improve the correct understanding of these results in the scientific community.

## Figures and Tables

**Table 1:** Baseline features of the whole sample were split into the two groups based on the use of RAASIs.

Variable	Non-Treated (n = 3557)	Treated (n = 1207)	*p*-Value	Correlation
Age, year	66.8± 14.5	64.5 ± 14.6	0.001	−0.071
Dialytic age, year	0 [0–1]	0 [0–1]	0.71	0.003
Sex, male	2178 (61)	782 (65)	0.028	0.032
Potassium, mmol/L	4.9 ± 0.8	5.0 ± 0.8	0.001	0.048
Albumin, g/dL	3.60± 0.50	3.64 ± 0.51	0.057	0.029
CRP, mg/dL	3.1 [1.1–7.3]	2.9 [1.0–6.1]	0.041	−0.041
Phosphate, mg/dL	4.7± 1.3	4.8 ± 1.3	0.010	0.038
Tsat, %	21.6± 12.5	22.7 ± 12.7	0.017	0.039
Iron, ng/mL	1665 (47)	641 (53)	0.001	0.055
Residual Diuresis, n(%)	1183 (33)	580 (48)	0.001	0.133
Cholesterol, mg/dL	158 ± 42	157 ± 42	0.620	−0.008
Use of ASA	1447 (41)	709 (59)	0.001	0.158
Use of a calcium antagonist	1236 (35)	697 (58)	0.001	0.204
Use of Folic acid	512 (14)	247 (20)	0.001	0.072
Use of Vit B12	158 (4)	86 (7)	0.001	0.053
Use of Β-blocker	1170 (33)	554 (46)	0.001	0.118
Use of Immunosuppressors	8 (0)	7 (1)	0.057	0.028
Arrhythmia, yes	139 (9)	44 (6)	0.008	−0.048
Arterial hypertension, yes	1546 (69)	660 (80)	0.001	0.171
Diabetes, yes	705 (32)	272 (36)	0.024	0.038
Heart failure, yes	228 (10)	50 (7)	0.003	−0.055
Liver chronic disease, yes	190 (9)	39 (5)	0.002	−0.056
COPD, yes	228 (10)	48 (6)	0.001	−0.056

ASA: Acetylsalicylic acid; CRP: C-reactive protein; COPD: Chronic obstructive pulmonary disease.

**Table 2:** Linear Mixed Model showing the direct association between the use of RAASIs and Serum potassium. β refers to the slope of the univariate LMM, whereas adjβ refers to the slope of the multivariate LMM.

	Longitudinal Univariate Random Intercept Analysis			Longitudinal Multivariate Analysis		
	β	95% CI	*p*	adjβ	95% CI	*p*
RAASIs, yes	0.05	0.03/0.07	*p* < 0.001	0.100	0.05/0.15	<0.001
Age, year				0.003	0.001/0.005	0.001
Sex, male				−0.011	−0.06/0.04	0.646
Albumin, g/dL				0.188	0.15/0.23	<0.001
Hypertension, yes				0.004	−0.05/0.06	0.124
Use of Calcium channel antagonists, yes				0.03	−0.008/0.07	0.122
COPD. yes				−0.081	−0.154/−0.008	0.03
CRP, g/dL				−0.002	−0.0033/−0.0006	0.014
Diabetes, yes				−0.087	−0.14/−0.03	0.001
Diuresis, yes				−0.116	−0.17/−0.06	<0.001
Serum Iron, ng/mL				−0.019	−0.05/0.01	0.199
heart failure, yes				−0.061	−0.14/0.02	0.151
serum phosphate, mg/dL				0.077	0.06/0.09	<0.001
Tsat, %				0.001	0.0003/0.0024	0.011
visit				0.011	0.008/0.015	<0.001
KT/V				0.034	−0.018/0.087	0.201

COPD: chronic obstructive pulmonary disease; CRP: C-Reactive Protein, RAASIs: renin-angiotensin-aldosterone system inhibitors, Tsat: Transferrin Saturation.

**Table 3:** Cox models showing the direct association between use of RAASIs and mortality.

	Cross-Sectional Univariate Analysis			Longitudinal Multivariate Analysis		
	HR	95%CI	*p*	HR	95%CI	*p*
RAASIs, yes	1.03	0.93/1.15	0.55	0.756	0.44/1.304	0.31
Serum Potassium, mmol/L				0.549	0.42/0.72	<0.001
Age, year				1.163	1.13/1.20	<0.001
Sex, male				1.359	0.72/2.60	0.35
Albumin, g/dL				0.350	0.23/0.54	<0.001
Hypertension, yes				0.598	0.29/1.26	0.17
arrhythmia. yes				1.468	0.75/2.84	0.026
Use of Antiaggregant, yes				0.975	0.60/1.60	0.92
Use of Calcium channel antagonists, yes				2.071	1.25/3.42	0.005
COPD, yes				2.259	1.02/5.00	0.045
CRP, mg/dL				1.012	0.998/1.026	0.095
Diabetes, yes				6.613	3.57/12.27	<0.001
Use of Folic acid, yes				0.689	0.39/1.22	0.2
heart failure, yes				7.751	3.40/17.69	<0.001
Visit, n				0.881	0.85/0.92	<0.001
KT/V				0.223	0.12/0.45	<0.001
Serum Phosphate, mg/dL				1.196	1.02/1.40	0.027

COPD: chronic obstructive pulmonary disease; CRP: C-Reactive Protein, RAASIs, renin-angiotensin-aldosterone system inhibitors.

## Data Availability

The data were retrieved from the Sicilian Registry of Nephrology, Dialysis, and Transplantation (http://www.crtsicilia.it/PUBLIC/RegistroRSNDT/CentriDialisiETx.aspx (accessed on 6 July 2022)).

## Supplementary Materials

The following supporting information can be downloaded at: https://www.mdpi.com/article/10.3390/jcm15134928/s1, Figure S1: Patient selection: Inclusion and Exclusion criteria.
