# Supplementary figures and images for: Impact of RAASIs on Potassium and Mortality in a Large Cohort of Hemodialysis Patients: Practical Excursus and Comparison Between Traditional Statistics and Machine Learning
Source: J Clin Med. 2026 Jun 25;15(13):4928. doi: 10.3390/jcm15134928 (PMC13361815; doi:10.3390/jcm15134928)

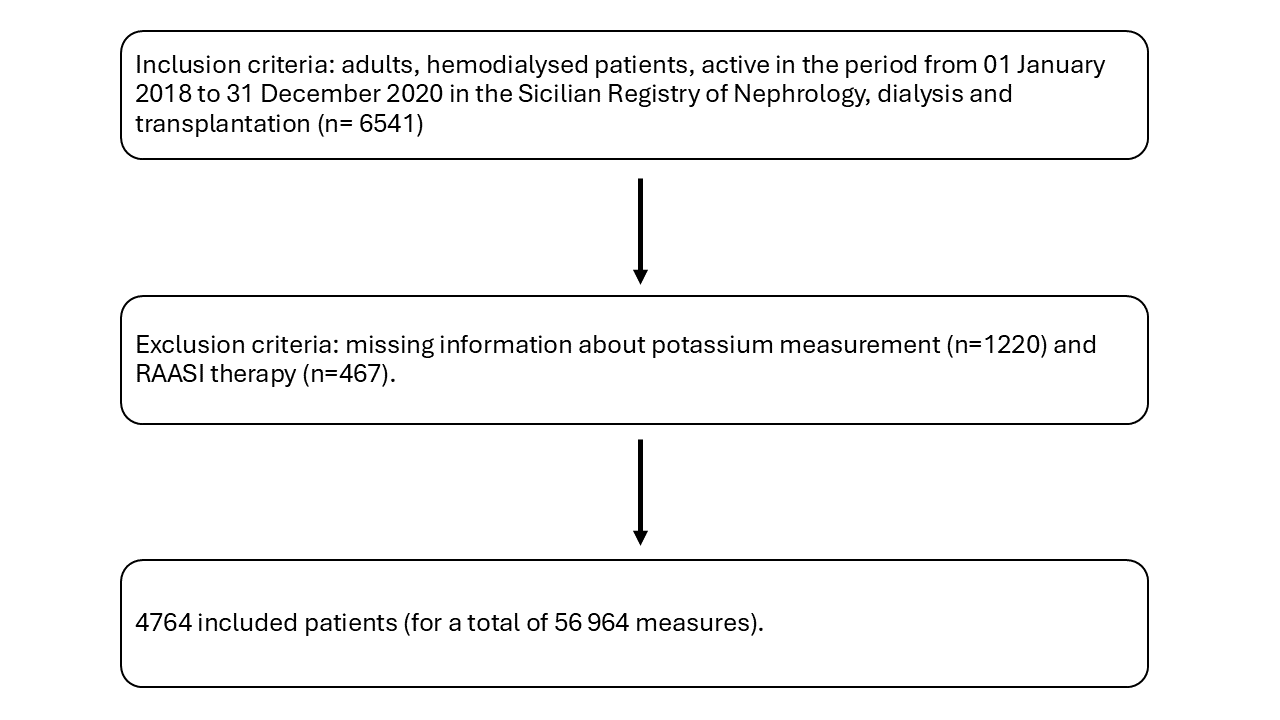

Supplement: Supplementary file 1 [file jcm-15-04928-s001.zip › jcm-4314553-supplementary.png]
